# Kiperin Mind Focus supplement mitigates chronic stress–induced neuroinflammation and molecular dysregulation and improves stress-related affective and exploratory behaviors in rats

**DOI:** 10.3389/fnint.2025.1745274

**Published:** 2026-01-29

**Authors:** Lutfiye Karcioglu Batur, Cuneyd Yavas, Aysu Kilic, Tunay Dogan, Mert Yilmaz, Ahsen Pektas, Huri Demirci, Savas Ustunova

**Affiliations:** 1 Department of Molecular Biology and Genetics, Faculty of Engineering and Natural Sciences, Biruni University, Istanbul, Türkiye; 2 Biruni University Research Center (B@MER), Biruni University, Istanbul, Türkiye; 3 Department of Physiology, School of Medicine, Bezmialem Vakif University, Istanbul, Türkiye; 4 Department of Medical Laboratory Techniques, Istinye University Vocational School of Health Care Services, Istanbul, Türkiye; 5 Department of Medical Biochemistry, School of Medicine, Biruni University, Istanbul, Türkiye

**Keywords:** adaptive exploratory behavior, BDNF, chronic stress, hippocampus, L-theanine, Mind Focus, neuroinflammation, neuroprotection

## Abstract

**Background:**

Chronic stress is known to impair emotional regulation and adaptive behavioral responses through neuroinflammatory activation, oxidative imbalance, and dysregulation of neuroplasticity-related genes. Kiperin Mind Focus, a nootropic nutraceutical containing L-theanine, citicoline, phosphatidylserine, *Rhodiola rosea, Ginkgo biloba*, caffeine, and Lion’s Mane mushroom extract has been formulated to support stress resilience, mood regulation and neural health. This study aimed to investigate the neuroprotective and neuroregulatory effects of the combined formulation on behavioral, biochemical, histopathological, and molecular parameters in rats exposed to chronic unpredictable mild stress (CUMS).

**Methods:**

Thirty-two adult male Wistar rats were randomized into four groups (*n* = 8): Control, Stress, Kiperin Mind Focus (MF), and Stress + Mind Focus (SMF). CUMS was applied for 45 days, and the combined formulation was administered by oral gavage (130 mg/kg/day). Behavioral outcomes were evaluated using the sucrose preference (SPT), open field (OFT), elevated plus maze (EPM), and forced swim (FST) tests. Serum and tissue cytokine levels (*IL-1*β*, IL-6, IL-10, TNF*-α) and oxidative stress index (TOS/TAS ratio) were measured. Hippocampal and prefrontal gene expression of *FOS, DBH, NMB, BDNF, CREB1, GRIN2A*, and *GABRB1* was assessed via qPCR, and histopathological changes were semi-quantitatively scored.

**Results:**

Chronic stress induced anhedonia, anxiety-like behavior, and behavioral despair, accompanied by elevated proinflammatory cytokines, oxidative imbalance, and neuronal degeneration in the hippocampus and prefrontal cortex. The supplementation significantly improved SPT, OFT, EPM, and FST performance, normalized cytokine and oxidative parameters, and reduced neuronal injury scores. At the molecular level, supplementation attenuated stress-induced upregulation of *FOS*, *DBH*, and *NMB* while maintaining neurotrophic (*BDNF*, *CREB1*) and GABAergic (*GABRB1*) expression near control levels.

**Conclusion:**

Kiperin Mind Focus exerted robust neuroprotective, anti-inflammatory, and antioxidant effects under chronic stress, restoring molecular homeostasis and stabilizing stress-related behavioral outcomes. These findings support its role as a stress-buffering and mood-stabilizing supplement, that promotes emotional regulation and adaptive exploratory behavior under prolonged stress conditions.

## Introduction

Optimal brain health is a multidimensional state encompassing cognitive, emotional, and motor domains supported by lifelong physiological processes that sustain neuronal integrity and mental performance (Chen et al., 2021). Cognitive functions—including attention, memory, executive control, and learning—can decline with age or chronic stress exposure, partly due to cumulative environmental and metabolic influences (American Psychiatric Association, 2013; Aubé, 2018). Age-related neuronal dysfunction and oxidative stress exacerbate this decline, whereas lifestyle and nutritional factors that enhance neuronal resilience may delay or prevent cognitive deterioration (Murman, 2015; Sergiev et al., 2015). As the global aging population increases, nutritional supplements targeting memory and focus have gained attention as adjunct strategies to support mental wellbeing and counteract stress-induced impairments in attention and cognition (Onaolapo et al., 2019).

Plant-derived bioactive compounds have long been recognized for their neuroprotective and adaptogenic properties (Al Akeel et al., 2018; Tamer et al., 2021). Nootropic agents—defined as substances that enhance cognitive performance, learning, and mental clarity—modulate neurotransmitter systems such as dopaminergic, glutamatergic, cholinergic, and serotonergic pathways (Onaolapo et al., 2019). Compared with synthetic psychostimulants, natural nootropics often exhibit fewer adverse effects while promoting neuronal plasticity and stress resilience. Among these, extracts such as Lion’s Mane mushroom, Rhodiola rosea, Ginkgo biloba, and compounds like L-theanine, citicoline, phosphatidylserine, and caffeine have demonstrated synergistic benefits in improving attention, mood, and neurochemical balance through modulation of neurotrophic factors, neurotransmission, and oxidative defense mechanisms (Adibhatla et al., 2002; Dager et al., 1999; Fredholm et al., 1999; Friedman, 2015; Ganzera et al., 2001; Gündoğdu, 2014; Jorissen et al., 2002; Lai et al., 2013; Marchev et al., 2016; Nguyen and Alzahrani, 2023; Wang et al., 2018; Wang et al., 2022; Weinmann et al., 2010).

Mind Focus (Kiperin Pharmaceutical and Food Industry Ltd., İstanbul, Turkiye) is a novel nutraceutical formulation that combines several well-established nootropic and adaptogenic ingredients—including L-theanine, Lion’s Mane mushroom extract, citicoline, phosphatidylserine, Rhodiola extract, caffeine, and Ginkgo biloba—designed to support stress resilience, mood regulation and overall neural health. Although each component has been individually investigated for its neuroprotective and psychotropic properties, their combined action on stress-related behavioral, biochemical, and transcriptional parameters have not been systematically examined. Therefore, the present study aimed to investigate the effects of supplementation on neural activity, neurochemical markers, gene expression profiles, and behavioral alterations in a chronic unpredictable mild stress (CUMS) rat model. Importantly, the behavioral assessment focused on stress-related affective and exploratory domains—including anhedonia, anxiety-like behavior, behavioral despair, and locomotor–exploratory activity—rather than classical attention or learning paradigms. Accordingly, the findings are intended to provide mechanistic insight into the potential behavioral, emotional and stress-buffering and behavioral regulatory effects of the combined formulation as a neuroactive nutritional supplement.

## Materials and methods

### Ethical approvals and animals

Thirty-two male *Wistar albino* rats, 3 months old, weighing 300–350 g were procured from Bezmialem Vakif University Experimental Animal Research Center. The animals were housed in groups of three to four per polycarbonate cage under standard laboratory conditions, with a controlled 12-h light/dark cycle, ambient temperature of 22 ± 1°C, and relative humidity maintained at approximately 60%. The rats were provided with *ad libitum* food and water. All experimental procedures complied with The National and Institutional guidelines for the ethical treatment of laboratory animals. The study protocol was approved by the Laboratory Animals Ethical Committee of Bezmialem Vakif University (No: 2025-8, Date: 26th Feb 2025). All animal experiments were conducted at the Bezmialem Vakif University Experimental Animal Research Center. Biochemical and molecular analyses were performed at Biruni University Research Center.

### Chemicals and experimental design

#### Chemicals

Mind Focus (Kiperin Pharmaceutical and Food Industry Ltd., İstanbul, Türkiye) contains the following active ingredients per capsule: L-Theanine (150 mg), Lion’s Mane mushroom extract (150 mg), Citicoline (125 mg), Phosphatidylserine (100 mg), Rhodiola extract (75 mg), Caffeine (75 mg), and Ginkgo biloba extract (45 mg). The rat dose was calculated based on the approved human daily dose of 1,440 mg for a 70-kg adult (equivalent to 20.6 mg/kg), as indicated by the Turkish Ministry of Agriculture and Forestry (Janhavi et al., 2019). Dose conversion from human to rat was performed using body surface area (BSA) normalization according to the Reagan–Shaw Km factor method, implemented via the DoseCal virtual dose conversion tool^1^ (Janhavi et al., 2019). Accordingly, the animal equivalent dose was calculated using the formula: Animal dose (mg/kg) = Human equivalent dose (mg/kg) × (Human Km / Rat Km), where Km values were 37 for humans and 6 for rats. This calculation yielded a rat-equivalent dose of approximately 127 mg/kg, which was rounded to 130 mg/kg. Mind Focus was therefore administered at 130 mg/kg/day, dissolved in 1 mL saline, by oral gavage for 30 days.

The total duration of the experiment was 45 days. Prior to the experiment, body weights were measured, and baseline sucrose preference tests were performed to assess hedonic status and ensure balanced group allocation. Rats were randomly assigned to four experimental groups (*n* = 8 per group):

1. Control group: The animals were housed under standard conditions with unrestricted access to food and water for 45 days without any stress. Subsequently, 1 ml of saline was administered by oral gavage for 30 days.
2. Stress group (CUMS): The animals were exposed to the CUMS model for 45 days. During this period, 1 ml of saline was administered by oral gavage for 30 days.
3. Mind Focus group: During this period, 130 mg/kg combined formulation, dissolved in 1 ml of saline, was administered by oral gavage for 30 days.
4. Stress + Mind Focus group: The animals were exposed to the CUMS model for 45 days. During this period, 130 mg/kg Mind Focus, dissolved in 1 ml of saline, was administered by oral gavage for 30 days.

### Chronic unpredictable mild stress protocol

The rats in stress groups were chronically exposed to various randomly scheduled stressors for 45 days adapted from of the procedure (Table 1; Willner et al., 2019). CUMS was applied daily from days 1 to 45. Mind Focus or saline administration by oral gavage was initiated on day 15 and continued for 30 consecutive days, overlapping with the latter phase of the CUMS protocol. Control rats were housed in a separate room to avoid indirect exposure to stress cues and were handled only for cage cleaning, weighing, and feeding. During stressor application (except for light disruption), animals were monitored every 30 min for signs of distress such as shivering, immobility, or lethargy. Animals showing atypical signs or injuries were removed from the stress protocol and evaluated by the attending veterinarian.

**TABLE 1:** Chronic unpredictable mild stress procedure.

Stressor	CUMS days
Hot plate (45°C, 5 min)	16, 28
Cold plate (5°C, 5 min)	6, 22, 40
Wet bedding (24 h)	14, 34, 41
Restraint stress (4 h)	7, 21, 35
Social isolation (24 h)	8, 19, 36
Cage tilting (45°, 24 h)	13, 25, 37
Tail cramping (1 min)	3, 15, 26, 32
Crowded housing (24 h)	9, 20, 38
Different cage (24 h)	10, 27, 39
Food deprivation (24 h)	1, 11, 29
Water deprivation (24 h)	2, 12, 18, 33
Reversal of day (24 h)	4, 24, 30
Reversal of night (24 h)	5, 17, 23, 31

**FIGURE 1:**
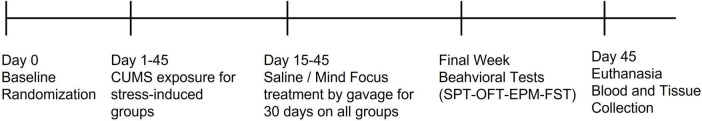
Experimental timeline of chronic unpredictable mild stress (CUMS) exposure and Mind Focus supplementation. Animals were randomized at baseline (day 0). CUMS was applied from days 1 to 45 in the Stress and Stress + Mind Focus groups. Mind Focus or saline was administered by oral gavage from days 15 to 45. Behavioral tests (SPT, OFT, EPM, and FST) were conducted during the final week, followed by euthanasia and tissue collection at day 45.

Behavioral assessments (SPT, OFT, EPM, and FST) were conducted during the final week of the experiment. All animals were euthanized on Day 45, and blood and brain tissues were collected at the experimental endpoint for biochemical, molecular, and histopathological analyses. A schematic representation of the experimental timeline is provided in Figure 1.

### Behavioral tests

#### Sucrose preference test

The sucrose preference test (SPT) was conducted three times throughout the experiment. Initially, it served to establish the basal anhedonia levels of the animals. Subsequently, tests were performed on the 13th day and at the conclusion of the experiment to verify anhedonia persistence. Before the first test, the animals were individually placed in single cages and accustomed to 1% sucrose solution in a quiet environment. Two bottles of 1% sucrose solution were initially placed in each cage. After 24 h, one sucrose bottle was replaced with drinking water. After the 24-h adaptation period, the animals were subjected to 24-h water and food deprivation. The next day, each cage received two bottles: one with 1% sucrose solution and the other with drinking water. Bottle positions were randomized after 1 h. At the end of 24 h, the weights of consumed sucrose solution and drinking water were recorded. The sucrose preference ratio (%) was calculated using the formula described in previous studies (Fonseca-Rodrigues et al., 2022; Hu et al., 2017).


SucrosePreferenceRate(%)=

Sucrose⁢Consumption⁢(g)Water⁢Consumption⁢(g)+Sucrose⁢Consumption⁢(g)⁢x⁢ 100

#### Open field test

Locomotor activity and exploratory behavior were measured in the open field test (OFT) (Zhang et al., 2020). The test apparatus had a square base (91 × 91 cm), was painted black, and had high walls (40 cm) to prevent the animal from climbing. The test apparatus was divided into a square-shaped middle area (20 × 20 cm) using the EthoVision-XT program. Following 5 min habituation, exploratory behavior was recorded for 5 min using an automated video tracking system (Noldus Information Technology, EthoVision System, Netherlands). The open-field apparatus was rinsed between sessions with 75% alcohol and dried with a towel to prevent any odor clues. During the 5-min session, the total distance traveled, time spent in the center area, transition frequency to the center area, locomotion latency, the frequency of rearing, and the time and frequency of grooming were measured. When evaluating the time spent in the center area, the presence of at least three extremities of rats was determined as a criterion.

#### Elevated plus maze

Anxiety-related exploration was assessed using the elevated plus-maze (EPM) by File and co-workers (Pellow et al., 1985). The test apparatus consists of a black wooden platform in the shape of a plus, consisting of two opposite open (50 × 10 × 40 cm) and two closed (50 × 10 × 40 cm) arms. The apparatus was elevated to a height of 50 cm above the floor. At the beginning of the experiment, each rat was placed in the middle compartment (5 × 5 cm) between the open and closed arms with the head facing toward an open arm and allowed to explore for 5 min. The maze was rinsed between sessions with 75% alcohol and dried with a towel to prevent any odor clues. During the experiment, the total time spent in the open arms (%) and closed arms (%) and entries into open arms (%) were recorded with the EthoVision-XT program (Noldus Information Technology, EthoVision System, Netherlands).

#### Forced swimming test

The forced swimming test (FST), also known as the behavioral despair test, focuses on a rodent’s response to the threat of drowning (Lino-de-Oliveira et al., 2005). Rats were forced to swim in a see-through Plexiglas cylinder that is 45 cm tall and 15 cm in diameter, filled with water (23–27°C, 35 cm deep) for 5 min. The entire experimental procedure was recorded with a camera. During the following 5 min, the duration of immobility time and latency to immobility for every rat were measured by two observers who were blinded to the kind of treatment.

### Biochemical analyses

#### Sample collections and homogenization

Blood samples were collected at baseline and at the end of the experiment. Serum was separated by centrifugation (3,000 rpm, 10 min) and stored at -80°C until analysis.

Liver, hippocampus and prefrontal cortex tissues were first washed in 0.9% NaCl and each tissue (∼50 mg) samples were placed on ice and transferred into a 2 mL screw-cap tube containing sterile zirconium beads (2.8 mm) and 1.0 mL of cold phosphate buffer (100 mM KH_2_PO_4_–K_2_HPO_4_, pH 7.4, containing 1.15% KCl) were added (w/v ∼1:10). Homogenization was performed using a bead−mill homogenizer with the following program: 5 m/s speed, 30 s per cycle, 3 cycles, with 30 s cooling on ice between cycles. After homogenization, the tubes were centrifuged at 10.000 × g for 10 min at 4°C and the supernatant collected for downstream analyses (Liang et al., 2011). Total protein concentration in the cleared homogenates was measured using the Bradford assay according to the method described by Bradford (1976).

#### Measurement of inflammatory cytokines

Frozen serum and tissue homogenates samples were thawed, and IL-6 (Cat No: ER0042), IL-10 (Cat No: ER0033), IL-1β (Cat No: ER1094) and TNF-α (Cat No: ER1393) levels were measured using commercial ELISA kits according to the manufacturers’ instructions. Briefly, samples and standards were added to wells which are pre-coated with related monoclonal antibodies. Biotin was added to all wells and combined with Streptavidin-HRP. The samples were incubated and washed for removing the uncombined enzymes. To stop the reaction, acid was added to the plates and optical density was measured at 450 nm via microplate reader (Thermo Scientific Microplate Reader, United States). The detection range of kits were between 15.625 and 2,000 pg/mL for IL-1β and IL-10, 31.25–4,000 pg/mL for IL-6, and 1.95–250 pg/mL for TNF-α.

#### Measurement of total antioxidant status and total oxidant status

Serum and tissues total TAS and TOS were determined with commercial kits (Rel Assay Diagnostics kit; Mega Tıp, Gaziantep, Türkiye) (Solakoglu et al., 2017). Additionally oxidative stress index (OSI) values were calculated according to the following formula: OSI = (TOS (μmol ⋅ H_2_O_2_ equivalent/g protein) / TAS (mmol.Trolox equivalent/g protein) X 100 (Erel, 2005). Briefly; TAS and TOS were measured in serum samples and tissue homogenates at wavelengths of 240 and 520 nm, respectively, using a plate reader (Thermo Scientific Multiskan FC, 2011-06, United States). For TAS measurements, Trolox, a water-soluble compound of vitamin E was used as a calibrator and the results were expressed as mmol. Trolox equivalent/L. For TOS measurements, H_2_O_2_ was used as the standard, and the results were expressed as μmol ⋅ H_2_O_2_ equivalent/L. OSI values were expressed as Arbitrary Units.

### Histopathology

From each animal, the hippocampus and prefrontal cortex were sampled, snap-frozen at –80°C and, at the time of histopathology, fixed in 10% neutral-buffered formalin. Tissues underwent routine processing (graded ethanol dehydration, xylene clearing, paraffin embedding) (Bancroft and Gamble, 2008); 5-μm paraffin sections were cut, mounted, deparaffinized/rehydrated, stained with hematoxylin–eosin (H&E), dehydrated, cleared, and coverslipped. Bright-field images were acquired at 20 × under constant exposure/white balance. Evaluation was blinded. A fixed, centrally placed square region of interest (ROI) of identical pixel size across images (calibrated at 20 × ) was analyzed per section. Exactly 20 pyramidal neurons within the ROI were inspected; k denotes the number that met “injured” criteria (red-neuron morphology: shrunken/triangular soma with hypereosinophilic cytoplasm and pyknotic nucleus; or ghost/shadow neuron: pale soma with no discernible nucleus). Perineuronal halo/vacuolization was visually estimated as percentage of the ROI. A 0–3 ordinal score was assigned as follows: 0 if k ≤ 1 and halo < 15%; 1 if k ≥ 2 and/or halo 15–30%; 2 if k = 5–9 and/or halo 30–60%; 3 if k ≥ 10 and/or halo > 60%.

### RNA isolation and relative expression levels evaluated by qPCR

Brain tissue samples were washed with PBS and stored at –80°C for RNA isolation. The samples were physically broken down by using a mortar and pestle and crushed in liquid nitrogen. Subsequently, samples were resuspended with TRIzol Reagent (Invitrogen, United States), and the total RNA isolation was performed according to the manufacturer’s protocol. Concentration and purity of the isolated RNA were assessed using a NanoDrop ND-2000c spectrophotometer (Thermo Fisher Scientific Inc., United States).

The relative expression levels of *FOS, DBH, NMB*, and *GRIN2A* were evaluated using quantitative real-time polymerase chain reaction (qPCR). cDNA synthesis was performed using 1,000 ng of total RNA with the OneScript^®^ Plus cDNA Synthesis Kit [Applied Biological Materials Inc. (Abm), Canada] according to the manufacturer’s instructions, in a T100 thermal cycler PCR machine (Bio-Rad, Singapore). qPCR experiments were conducted in duplicate using the BlasTaq™ 2X qPCR Master Mix (Abm, Canada) and the LightCycler 480 instrument (Roche, Germany). *GAPDH* was used as the endogenous control for normalization of gene expression. The primers used were as follows: for *FOS*, forward 5’- TACTACCATTCCCCAGCCGA-3’and reverse 5’-GCTGTCACCGTGGGGATAAA-3’; for *DBH*, forward 5’-CCTTCCCCATGTTCAACGGA-3’ and reverse 5’- ACCGGCTTCTTCTGGGTAG-3’; for *NMB*, forward 5’- CCCAGAGGGAGCAGAGACTA-3’ and reverse 5’-TGGACCACTGAGGTTCATGC-3’; for *GRIN2A*, forward 5’- CCCAGGCTTGTGGTGATCGT-3’ and reverse 5’-CGAAG GGGGCTTCCTCCAAG-3’ for *GAPDH* (housekeeping gene), forward 5’-TTCACCACCATGGAGAAGGC-3’ and reverse 5’-CTCGTGGTTCACACCCATCA-3’. Relative gene expression levels were calculated using the 2^–ΔΔCt^ method. All reactions included no-template controls to assess contamination and nonspecific amplification.

### Statistical analysis

All statistical analyses were performed using GraphPad Prism 8 (GraphPad Software Inc., San Diego, CA, United States) and R version 4.5.1 with the ggplot2 (v4.0.0), FSA (v0.10.0), ggsignif (v0.6.4), and effsize (v0.8.1) packages. Data distribution was assessed for normality using the Kolmogorov–Smirnov test. For normally distributed data, comparisons among the four independent experimental groups were conducted using one-way analysis of variance (ANOVA) followed by the Tukey–Kramer multiple comparison *post hoc* test. For non-normally distributed data, the Kruskal–Wallis test with Dunn’s *post hoc* analysis and Holm correction was applied. Descriptive statistics are presented as mean ± standard deviation (SD).

No formal statistical outlier detection tests (e.g., Grubbs’ test or ROUT method) were applied to behavioral, biochemical, or gene expression datasets. All data points obtained from individual animals were retained and included in the final analyses. To account for potential variability and small group sizes, data distribution and variance assumptions were evaluated using Brown–Forsythe and Bartlett’s tests, and Welch’s correction was applied when appropriate. Non-parametric tests were used where distributional assumptions were not met. Analyses were therefore conducted without exclusion of extreme values, ensuring that the reported results reflect the full biological variability of the experimental groups. A *p* < 0.05 was considered statistically significant for all analyses. Given the number of molecular endpoints assessed across multiple tissues, the gene expression analyses were considered exploratory in nature, and results—particularly those with marginal *p*-values—should be interpreted with caution due to the increased risk of type I error associated with multiple comparisons.

## Results

### Body weight

**FIGURE 2:**
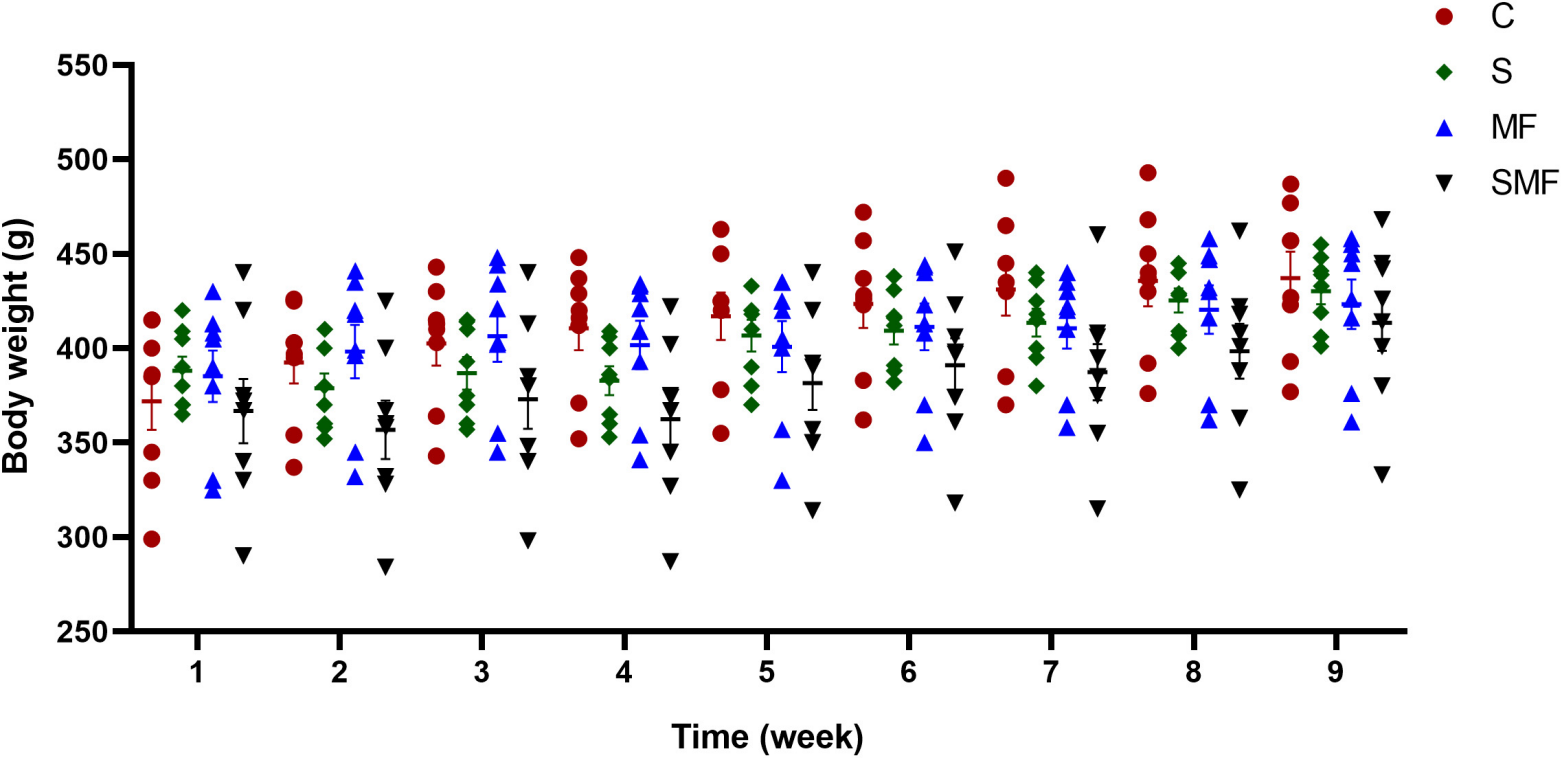
Changes in body weight over the experimental period. Weekly body weight measurements of rats in the control (C), stress (S), Mind Focus (MF), and stress + Mind Focus (SMF) groups throughout the 9-week experimental period. Data is presented as mean ± SD.

Throughout the experimental period, body weight was regularly monitored (Figure 2). Body weights of all groups significantly increased during the 7-week experimental period (*p* < 0.001). In contrast, treatment with MF either alone or in combination with stress, maintained body weights comparable to controls, indicating that supplementation may mitigate stress-induced weight loss.

### Sucrose preference test

**FIGURE 3:**
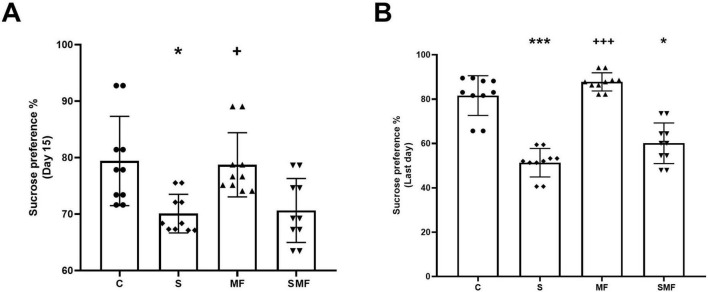
Sucrose preference test results. **(A)** Sucrose preference percentage on day 15 and **(B)** on the last day of the experiment in control **(C)**, stress (S), Mind Focus (MF), and stress + Mind Focus (SMF) groups. Data are presented as mean ± SD. *p < 0.05, ***p < 0.001 compared to control group; **^+^***p* < 0.05, **^+++^***p* < 0.001 compared to S group.

The sucrose preference test was performed to assess anhedonic behavior. Rats exposed to the CUMS protocol displayed a significant reduction in sucrose preference compared to controls (*p* < 0.01) at the 13th day of the CUMS period, indicating the development of anhedonia (Figure 3A). Sucrose preference significantly decreased in the S (*p* < 0.001) and SMF (*p* < 0.05) groups than in the controls and significantly increased in the MF group compared to the S group (*p* < 0.001) (Figure 3B).

### Open field test

**FIGURE 4:**
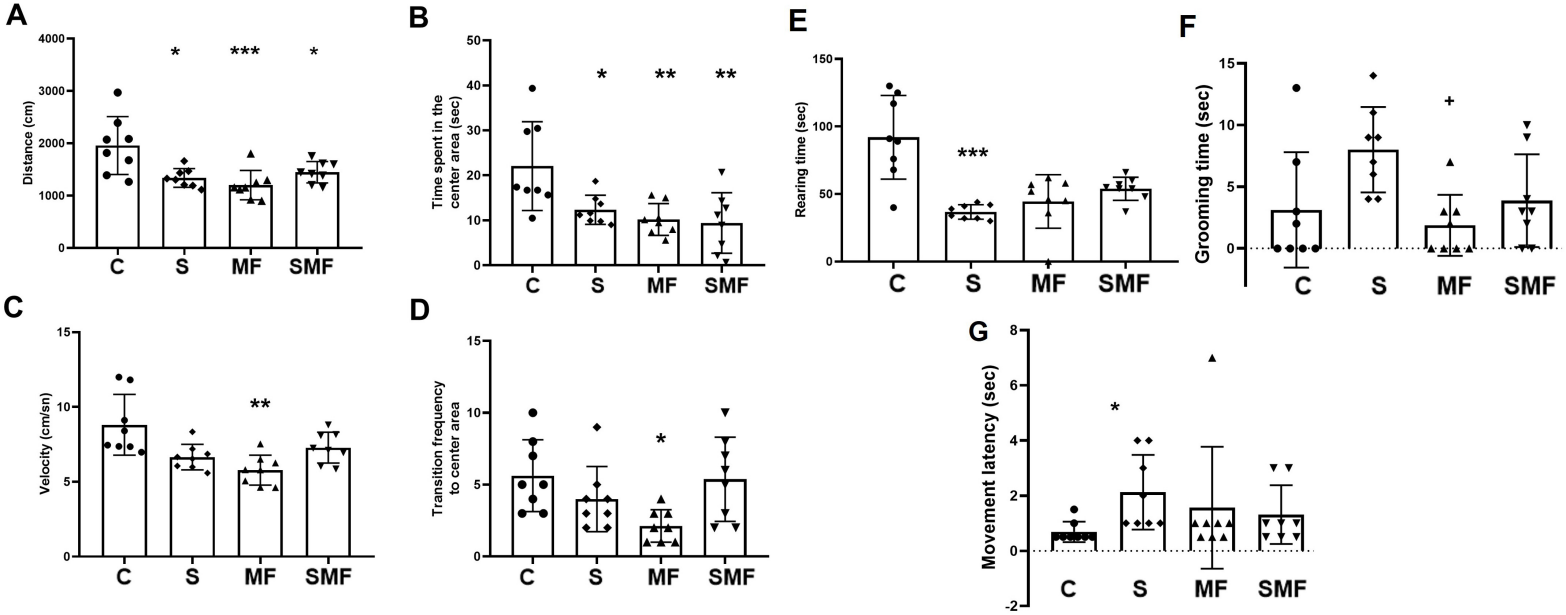
Open field test results. **(A)** Total distance traveled (cm), **(B)** time spent in the center zone (s), **(C)** velocity (cm/s), **(D)** transition frequency to the center zone **(E)** rearing time (s), **(F)** grooming time (s), and **(G)** movement latency (s) in the control (C), stress (S), Mind Focus (MF), and stress + Mind Focus (SMF) groups. Data are presented as mean ± SD. *p < 0.05, **p < 0.01, ***p < 0.001 compared to control group; **^+^***p* < 0.1 compared to S group.

Open field test was performed to measure animals’ anxiety levels and locomotor activity. Total distance was significantly increased in the S (*p* < 0.05), MF (*p* < 0.001) and SMF (*p* < 0.05) groups compared to the controls (Figure 4A). Similarly, time spent in the center area was significantly increased in the SMF (*p* < 0.05), MF (*p* < 0.001) and SMF (*p* < 0.001) groups than in the controls (Figure 4B). The lowest values of velocity (*p* < 0.01) (Figure 4C) and transition frequency to the center area (*p* < 0.05) (Figure 4D) were detected in the MF group compared to the controls.

Rearing time significantly decreased in the S group (*p* < 0.001) than in the control group (Figure 4E). Additionally, grooming time of animals significantly decreased in the MF group (*p* < 0.05) than in the S group (Figure 4F). Lastly, movement latency of animals significantly increased in the S group (*p* < 0.05) compared to the controls (Figure 4G).

### Elevated plus maze

**FIGURE 5:**
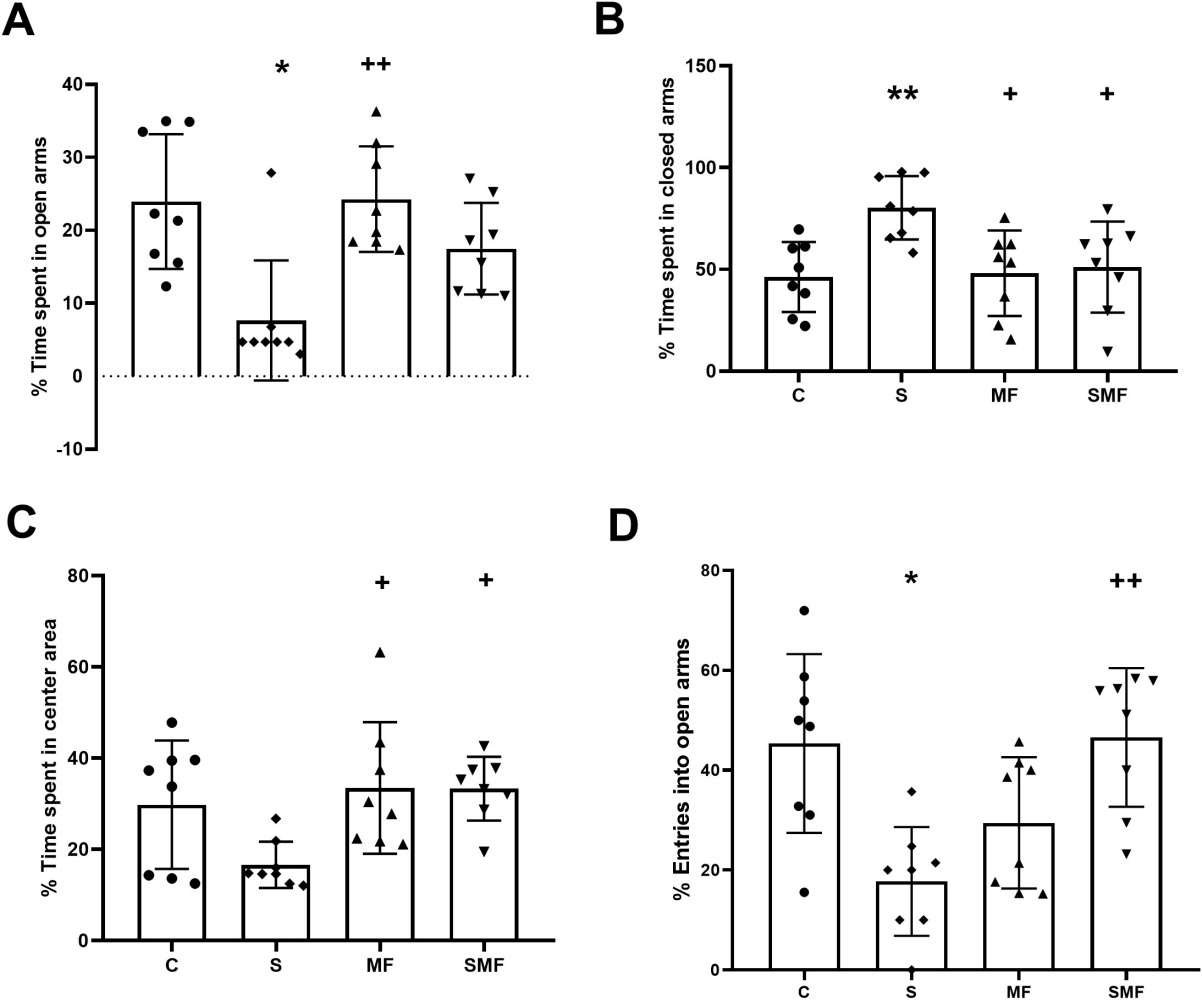
Elevated Plus Maze test results. **(A)** Percentage of time spent in the open arms, **(B)** percentage of time spent in the closed arms, **(C)** percentage of time spent in the center area, and **(D)** percentage of entries into the open arms in the control (C), stress (S), Mind Focus (MF), and stress + Mind Focus (SMF) groups. Data are presented as mean ± SD. *p < 0.05, **p < 0.01 compared to control group; **^+^***p* < 0.05, **^++^***p* < 0.01 compared to S group.

In the EPM, time spent in open arms of animals significantly decreased (*p* < 0.05) in the S group compared to the controls and significantly increased (*p* < 0.01) in the MF group than in the S group (Figure 5A). To support this finding, time spent in closed arms of animals significantly increased in the S group (*p* < 0.01) than in the control group and significantly decreased (*p*’s < 0.05) in MF and SMF groups compared to the S group (Figure 5B). Time spent in the center areas significantly increased (*p*’s < 0.05) in both MF and SMF groups than in the S group (Figure 5C). Lastly, entries into open arms significantly decreased (*p* < 0.05) in the S group compared to the controls and significantly increased (*p* < 0.01) in the SMF group than in the S group (Figure 5D).

### Forced swim test

**FIGURE 6:**
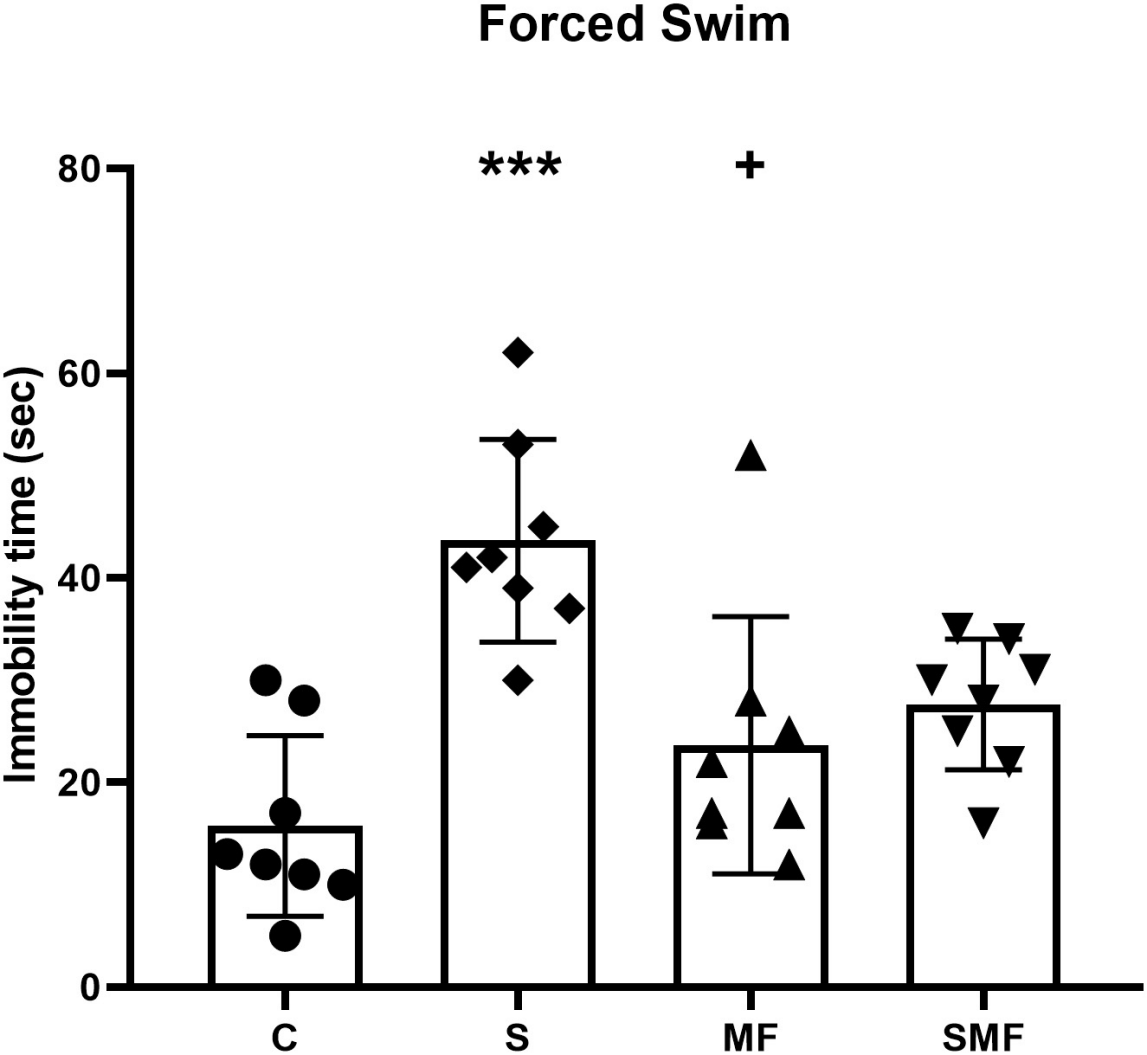
Forced Swim Test results. Immobility time (s) during the Forced Swim Test in the control (C), stress (S), Mind Focus (MF), and stress + Mind Focus (SMF) groups. Data are presented as mean ± SD. ***p < 0.001 compared to control group; **^+^***p* < 0.05 compared to S group.

In the forced swim test, rats subjected to chronic stress exhibited a significant increase in immobility duration compared to controls (*p* < 0.01), indicating a depression-like phenotype. Rats in the MF group showed significantly shorter immobility times compared to S group (*p* < 0.05), suggesting an antidepressant-like effect of the supplement under chronic stress conditions (Figure 6).

### Quantitative levels of inflammation biomarkers

**FIGURE 7:**
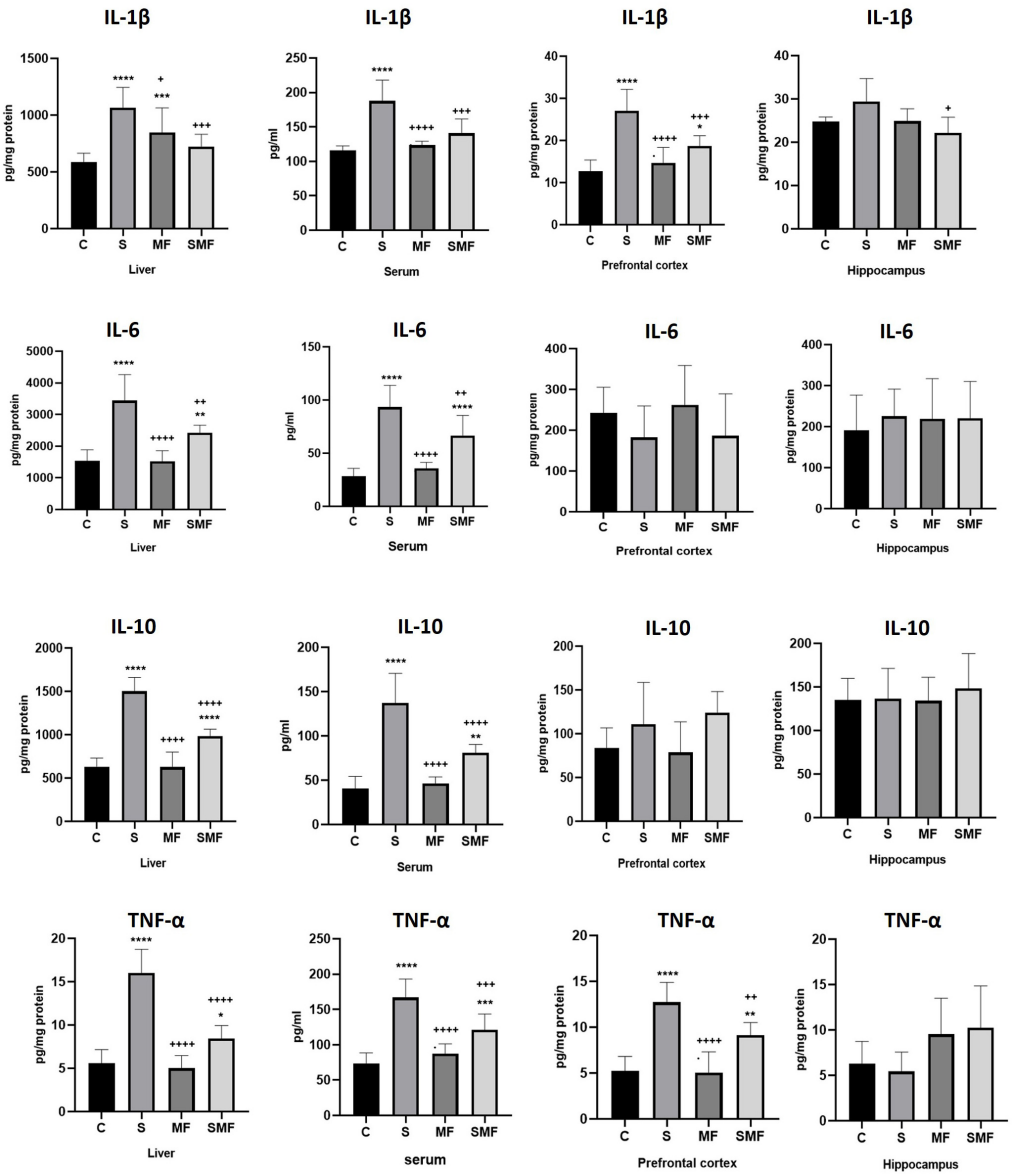
Quantitative levels of inflammation markers IL-1β, IL-6, IL-10 and TNF-α in serum, liver, prefrontal cortex and hippocampus. C, control group; S, stress group; MF, Mind Focus group; SMF, Stress+ Mind Focus group; *p < 0.05, **p < 0.005, ***p < 0.0005, and **** *p* < 0.0001 vs. control group; + *p* < 0.05, ++ *p* < 0.005, +++*p* < 0.0005 and ++++ *p* < 0.0001 vs. stress group.

Quantitative assessment of proinflammatory and anti-inflammatory cytokines revealed distinct systemic and regional patterns across the experimental groups (Figure 7). Chronic stress markedly elevated IL-1β levels in the liver, serum, and prefrontal cortex compared with the control group (*p* < 0.0001), indicating pronounced systemic and neuroinflammatory activation. In contrast, hippocampal IL-1β levels showed a mild but non-significant increase. Mind Focus treatment effectively mitigated these stress-induced elevations. Both MF and SMF significantly reduced hepatic and serum IL-1β levels compared with the stress group (*p* < 0.05 for all), while maintaining values comparable to controls. In the prefrontal cortex, MF treatment normalized IL-1β expression to near-control levels, and SMF administration also significantly attenuated the stress-induced increase (*p* < 0.001).

Similar to IL-1β, IL-6 concentrations were significantly increased in the liver and serum of stressed animals (*p* < 0.0001), reflecting systemic inflammatory activation (Figure 7). However, IL-6 levels in the prefrontal cortex and hippocampus remained unchanged across groups (*p* > 0.05). Both MF and SMF treatments significantly reduced hepatic and serum IL-6 levels compared to the stress condition (*p* < 0.01), with the MF group showing slightly stronger normalization. These findings indicate that the supplement primarily mitigated peripheral IL-6 upregulation associated with chronic stress.

In peripheral tissues, IL-10 levels were also significantly elevated in the stress group (*p* < 0.0001), suggesting compensatory anti-inflammatory activation (Figure 7). MF administration maintained IL-10 concentrations comparable to controls, whereas SMF treatment resulted in a moderate but significant increase. Nevertheless, both treatment groups displayed markedly lower IL-10 levels than the stress group (*p* < 0.0001), indicating effective modulation of peripheral immune balance. In the prefrontal cortex and hippocampus, IL-10 levels remained stable without significant changes among groups (*p* > 0.05).

Chronic stress exposure significantly increased TNF-α levels in the liver, serum, and prefrontal cortex (*p* < 0.0001), while hippocampal concentrations were less affected (Figure 7). Mind Focus administration effectively attenuated this proinflammatory response. MF treatment maintained TNF-α levels comparable to controls across all tissues, whereas SMF showed mild but significant elevations relative to control values. However, both MF and SMF treatments produced significantly lower TNF-α levels than the stress group (*p* < 0.001), confirming a substantial anti-inflammatory effect.

### Quantitative levels of oxidative stress indexes

**FIGURE 8:**
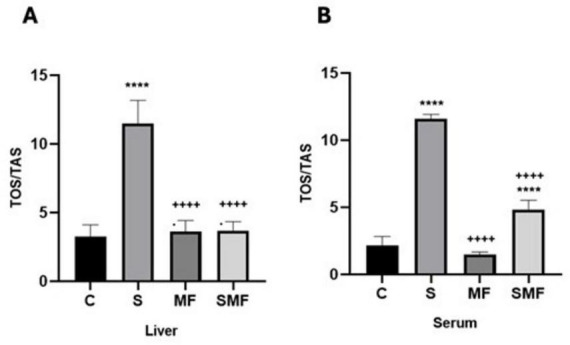
Quantitative levels of oxidative stress indexes (TAS/TOS) in liver tissue **(A)** and serum **(B)** of the control (C), stress (S), Mind Focus (MF), and stress + Mind Focus (SMF) groups. ****p < 0.0001 vs. control group; ++++*p* < 0.0001 vs. stress group.

OSI values in liver and serum revealed distinct alterations among the experimental groups (Figure 8). In both tissues, chronic stress produced a marked elevation in OSI compared with the control group (*p* < 0.0001), indicating a clear shift toward oxidative imbalance under stress conditions. MF treatment alone did not significantly differ from control values (*p* > 0.05) in either liver or serum, while SMF exposure yielded a notable decrease in OSI relative to the stress group (*p* < 0.0001). Both treatment groups exhibited substantially lower OSI values compared with the stress condition (*p* < 0.0001 for all comparisons). A significant difference between the MF and SMF groups was also observed in serum samples (*p* < 0.0001), whereas their hepatic OSI values remained comparable (*p* > 0.05).

### Histopathological findings

**FIGURE 9:**
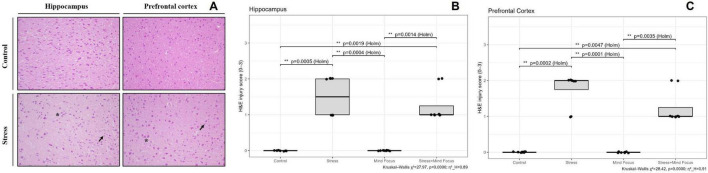
**(A)** Representative H&E micrographs of the hippocampus (left) and prefrontal cortex (right) from Control and Stress groups (20×). Control sections show preserved neuronal morphology. In stressed animals, perineuronal halo/spongiosis is evident (asterisks) and triangular, hypereosinophilic shrunken neurons (“red neuron–like”; arrows) are occasionally observed. **(B)** Hippocampus—semi-quantitative H&E injury scores (0–3) across groups (Control, Stress, Mind Focus, Stress+Mind Focus). **(C)** Prefrontal cortex—semi-quantitative H&E injury scores (0–3) across groups (Control, Stress, Mind Focus, Stress+Mind Focus). Each dot represents one mouse (*n* = 8/group). Boxes show median and interquartile range; whiskers denote 1.5 × IQR. Pairwise contrasts were tested with Dunn’s test (Holm correction); brackets display Holm-adjusted *p*-values (*p < 0.05, **p < 0.005). While some freezing-related artifacts (e.g., perineuronal halos or dark neurons) may be present, the images illustrate consistent group-specific differences in neuronal integrity and tissue architecture evaluated using the same scoring criteria across all groups.

Figure 9 represents the micrographs of hippocampus and prefrontal cortex from control and stress induced groups. Control sections showed preserved neuronal morphology while the sections from stressed animals indicated evident and triangular perineuronal halo/spongiosis, and occasional hypereosinophilic shrunken neurons (Figure 9A). Across groups (*n* = 8 per group), semi-quantitative H&E injury scores differed significantly in both regions (hippocampus: Kruskal–Wallis χ^2^ = 27.97, *p* < 0.001; prefrontal cortex: χ = 28.42, *p* < 0.001; η_H≈0.89–0.91). Stress increased scores vs. Control (Holm-adjusted *p* ≤ 0.0005), whereas Mind Focus alone did not differ from Control (all 0). Importantly, Stress+Mind Focus showed lower scores than Stress in the hippocampus (*p* = 0.0019) and prefrontal cortex (*p* = 0.0047), indicating a protective effect under stress (Figures 9B,C).

### Hippocampal gene expression

Quantitative analysis of hippocampal gene expression revealed statistically significant stress-related alterations in *FOS, DBH, NMB*, and *GABRB1*, whereas changes in *BDNF, CREB-1*, and *GRIN2A* did not reach statistical significance across groups (Table 2). Hippocampal *FOS* expression differed significantly among groups (overall *p* = 0.001). *Post-hoc* analysis demonstrated a significant upregulation in the stress group compared with controls (C vs. S, *p* = 0.035). Both MF and SMF groups exhibited significantly lower FOS expression relative to the stress group (S vs. MF, *p* < 0.001; S vs. SMF, *p* = 0.008), while neither treatment group differed significantly from controls (all *p* > 0.05), indicating attenuation of stress-induced FOS upregulation.

**TABLE 2:** Relative hippocampal expression levels of *FOS*, *DBH*, *NMB*, *BDNF, CREB-1, GRIN2A* and *GABRB1* across experimental groups.

Groups	Control (C)	Stress (S)	Mind focus (MF)	Stress+mind focus (SMF)	*P*-value	*Post-hoc*	*p*-values
*FOS*	0.91 ± 0.66	1.48 ± 1.11	0.66 ± 0.19	0.80 ± 0.19	**0.001**	C vs. S	**0.035**
						C vs. MF	0.446
						C vs. SMF	0.707
						S vs. MF	**< 0.001**
						S vs. SMF	**0.008**
						MF vs. SMF	0.707
*DBH*	1.08 ± 0.48	1.92 ± 1.02	1.32 ± 1.03	1.45 ± 0.80	**0.017**	C vs. S	**0.011**
						C vs. MF	0.819
						C vs. SMF	0.557
						S vs. MF	0.107
						S vs. SMF	0.318
						MF vs. SMF	0.964
*NMB*	1.12 ± 0.61	2.13 ± 1.18	1.52 ± 1.32	1.65 ± 0.62	**0.041**	C vs. S	**0.002**
						C vs. MF	0.211
						C vs. SMF	**0.030**
						S vs. MF	0.228
						S vs. SMF	0.560
						MF vs. SMF	0.985
*BDNF*	8.83 ± 3.50	6.47 ± 1.12	7.73 ± 0.67	6.82 ± 0.99	0.106	C vs. S	0.102
						C vs. MF	0.723
						C vs. SMF	0.199
						S vs. MF	0.579
						S vs. SMF	0.979
						MF vs. SMF	0.792
*CREB-1*	1.24 ± 0.15	0.93 ± 0.38	1.16 ± 0.40	1.33 ± 0.40	0.263	C vs. S	0.446
						C vs. MF	0.979
						C vs. SMF	0.967
						S vs. MF	0.619
						S vs. SMF	0.228
						MF vs. SMF	0.812
*GRIN2A*	1.19 ± 0.35	1.50 ± 0.19	1.46 ± 0.99	1.08 ± 0.81	0.670	C vs. S	0.866
						C vs. MF	0.917
						C vs. SMF	0.994
						S vs. MF	> 0.999
						Svs. SMF	0.708
						MF vs. SMF	0.794
*GABRB1*	27.42 ± 0.76	14.60 ± 7.68	18.57 ± 5.80	17.39 ± 1.97	**0.033**	C vs. S	**0.025**
						C vs. MF	0.168
						C vs. SMF	0.100
						S vs. MF	0.740
						S vs. SMF	0.889
						MF vs. SMF	0.990

The bold values indicate statistically significant *p*-values.

Similarly, hippocampal *DBH* expression showed a significant overall group effect (*p* = 0.017), with increased expression in the stress group compared to controls (C vs. S, *p* = 0.011). *DBH* expression in the MF and SMF groups did not differ significantly from either the control or stress groups (all *p* > 0.05), suggesting no statistically significant normalization, despite numerically lower mean values relative to the stress group.

Hippocampal *NMB* expression was significantly altered across groups (*p* = 0.041). The stress group displayed markedly higher NMB expression than controls (C vs. S, *p* = 0.002). While MF treatment alone did not significantly affect NMB expression compared to the stress group (*p* = 0.228), the SMF group showed significantly lower expression relative to controls (C vs. SMF, *p* = 0.030), whereas no significant difference was observed between the stress and SMF groups (*p* = 0.560), indicating partial modulation rather than full normalization.

In contrast, hippocampal *BDNF*, *CREB-1*, and *GRIN2A* expression levels did not differ significantly among experimental groups (all overall *p* > 0.05). Although mean values for BDNF and *CREB-1* were numerically reduced in the stress group and appeared closer to control levels in MF-treated groups, these changes did not reach statistical significance and were therefore considered non-significant trends rather than demonstrable effects.

Hippocampal *GABRB1* expression showed a significant overall group difference (*p* = 0.033), with a marked reduction in the stress group compared to controls (C vs. S, *p* = 0.025). Neither MF nor SMF groups differed significantly from the stress group or controls (all *p* > 0.05), indicating that supplementation did not statistically restore hippocampal *GABRB1* expression, despite intermediate mean values.

### Prefrontal gene expression

Analysis of prefrontal cortex gene expression identified statistically significant stress-related changes in *DBH, BDNF*, and *CREB-1*, whereas *FOS, NMB, GRIN2A*, and *GABRB1* expression remained unchanged across groups (Table 3). Prefrontal DBH expression differed significantly across groups (overall *p* = 0.042). *Post-hoc* comparisons revealed significantly lower DBH expression in the MF group compared with the stress group (S vs. MF, *p* = 0.035), while no significant differences were observed between the stress and control groups or between the SMF and control groups (all *p* > 0.05).

**TABLE 3:** Relative prefrontal expression levels of *FOS*, *DBH*, *NMB*, *BDNF, CREB-1, GRIN2A* and *GABRB1* across experimental groups.

Groups	Control (C)	Stress (S)	Mind focus (MF)	Stress+mind focus (SMF)	*P*-value	*Post-hoc*	*p*-values
*FOS*	0.78 ± 0.12	0.92 ± 0.25	0.80 ± 0.68	0.85 ± 0.20	0.940	C vs. S	0.949
						C vs. MF	> 0.999
						Cvs. SMF	0.995
						S vs. MF	0.951
						S vs. SMF	0.990
						MF vs. SMF	0.997
*DBH*	0.97 ± 0.14	1.05 ± 0.32	0.65 ± 0.24	0.98 ± 0.21	**0.042**	C vs. S	0.913
						C vs. MF	0.118
						C vs. SMF	> 0.999
						Svs. MF	**0.035**
						S vs. SMF	0.932
						MF vs. SMF	0.091
*NMB*	0.96 ± 0.52	1.59 ± 0.79	0.87 ± 0.19	0.89 ± 0.20	0.078	C vs. S	0.144
						C vs. MF	0.987
						C vs. SMF	0.989
						S vs. MF	0.111
						S vs. SMF	0.079
						MF vs. SMF	> 0.999
*BDNF*	0.99 ± 0.37	0.46 ± 0.05	0.66 ± 0.19	0.87 ± 0.32	**0.005**	C vs. S	**0.004**
						C vs. MF	0.217
						C vs. SMF	0.787
						S vs. MF	0.628
						S vs. SMF	**0.029**
						MF vs. SMF	0.599
*CREB-1*	0.97 ± 0.36	0.53 ± 0.10	0.65 ± 0.21	0.72 ± 0.35	**0.047**	C vs. S	**0.034**
						C vs. MF	0.181
						C vs. SMF	0.382
						S vs. MF	0.883
						S vs. SMF	0.627
						MF vs. SMF	0.967
*GRIN2A*	1.06 ± 0.35	0.82 ± 0.36	1.01 ± 0.20	1.04 ± 0.37	0.767	C vs. S	0.518
						C vs. MF	0.991
						C vs. SMF	> 0.999
						Svs. MF	0.744
						S vs. SMF	0.663
						MF vs. SMF	0.998
*GABRB1*	1.03 ± 0.25	0.97 ± 0.17	1.01 ± 0.39	0.87 ± 0.06	0.700	C vs. S	0.987
						C vs. MF	> 0.999
						Cvs. SMF	0.691
						S vs. MF	0.994
						S vs. SMF	0.908
						MF vs. SMF	0.742

The bold values indicate statistically significant *p*-values.

Prefrontal *BDNF* expression demonstrated a robust overall group effect (*p* = 0.005). *BDNF* levels were significantly reduced in the stress group compared with controls (C vs. S, *p* = 0.004). In contrast, the SMF group exhibited significantly higher BDNF expression than the stress group (S vs. SMF, *p* = 0.029), while MF alone did not differ significantly from either controls or stressed animals (all *p* > 0.05), indicating a statistically supported restoration of stress-induced *BDNF* suppression in the combined treatment condition.

Similarly, prefrontal *CREB-1* expression differed significantly among groups (*p* = 0.047), with significantly lower expression in the stress group compared with controls (C vs. S, *p* = 0.034). *CREB-1* expression in the MF and SMF groups did not differ significantly from either the control or stress groups (all *p* > 0.05), suggesting partial but statistically non-significant recovery.

Expression levels of prefrontal *FOS, NMB, GRIN2A*, and *GABRB1* did not differ significantly among groups (all overall *p* > 0.05). Although mean values in MF-treated groups were numerically closer to control levels for some genes, these differences were not statistically significant and were therefore interpreted as non-significant trends.

## Discussion

The present study demonstrated that Mind Focus supplementation counteracted CUMS-induced affective disturbances and modulated exploratory activity, as reflected by improvements in anhedonia- (SPT), anxiety-like behavior– (EPM), and behavioral despair–related (FST) measures, together with OFT readouts of locomotion and exploration. Importantly, because no dedicated attention or learning tasks were included (e.g., novel object recognition, set-shifting, 5-CSRTT), these behavioral findings should not be interpreted as direct evidence of enhanced attention or focus. Rather, the observed behavioral changes improved stress-related emotional regulation, behavioral engagement, and exploratory activity under chronic stress conditions. Taken together, these findings suggest that the combined formulation may exert its effects by modulating neural processes associated with affective regulation, arousal, and adaptive behavioral responses, rather than directly enhancing attentional or cognitive performance.

At the molecular and biochemical level, Mind Focus supplementation mitigated the wide-ranging effects of chronic stress on neuroinflammation, oxidative balance, and synaptic signaling. The stress-induced elevations of proinflammatory cytokines (IL-1β, IL-6, and TNF-α) in serum, liver, and prefrontal cortex were markedly attenuated by this supplementation, restoring peripheral and central cytokine levels to near-control values. In parallel, the oxidative stress index (TOS/TAS ratio) was significantly reduced in both serum and liver following treatment, indicating improved redox homeostasis. Gene expression analyses revealed that the combined formulation modulated several molecular markers of neural activation (*FOS*), catecholaminergic signaling (*DBH*), and neuropeptidergic regulation (*NMB*), while preserving neuroplasticity-related transcripts such as *BDNF, CREB1, GRIN2A*, and *GABRB1* in both hippocampal and prefrontal regions. Notably, the partial normalization of *BDNF* and *CREB1* expression suggests restoration of neurotrophic support, whereas the stabilization of *GABRB1* points to recovery of inhibitory tone, aligning with the observed behavioral improvements.

In the sucrose preference test, Mind Focus supplementation alleviated stress-induced anhedonia, reflecting antidepressant-like effects and improved hedonic motivation under chronic stress. This behavioral improvement is likely mediated by the synergistic activity of its neuroactive constituents that collectively modulate monoaminergic transmission, neurotrophic signaling, and stress hormone regulation. Because we tested only the combined formulation, we cannot attribute these effects to any single ingredient; however, prior studies suggest that L-theanine, a green tea–derived amino acid, has been shown to reverse CUMS-induced depressive-like behaviors by increasing serotonin, dopamine, and norepinephrine levels in limbic–cortical regions (Shen et al., 2019), while citicoline enhances antidepressant efficacy and sucrose preference in stressed rodents through its effects on serotonergic neurotransmission and membrane phospholipid dynamics (Roohi-Azizi et al., 2018). Rhodiola rosea extract, a well-known adaptogen, reduces corticosterone elevation and regulates stress-responsive gene expression in the hippocampus and prefrontal cortex, contributing to enhanced stress resilience (Dinel et al., 2019). Similarly, Ginkgo biloba extract (EGb 761) has been reported to counteract LPS-induced anhedonia and restore dopamine levels in the nucleus accumbens, suggesting a regulatory effect on reward-related pathways (Yeh et al., 2015). In addition, Lion’s Mane mushroom extract contains bioactive compounds such as erinacines and hericenones that promote neurogenesis and synaptic plasticity; while its acute effects on cognition appear domain-specific, improvements in psychomotor performance and attentional engagement observed in healthy adults (Surendran et al., 2025), support its potential to modulate mood and stress resilience. Collectively, prior evidence suggests that several components of the nutraceutical formulation may contribute to the restoration of sucrose preference in treated rats which may reflect a multifaceted enhancement of dopaminergic and serotonergic signaling, neurotrophic activity, and HPA axis regulation, jointly contributing to improved motivation, emotional balance, and exploratory activity control under stress conditions.

In the open field test, Mind Focus supplementation significantly enhanced exploratory activity and reduced anxiety-like behavior in rats subjected to chronic stress, indicating improved emotional regulation and behavioral control. This anxiolytic effect is consistent with the known actions of L-theanine, which has been shown to reverse chronic restraint stress–induced behavioral alterations by normalizing prefrontal neurotransmitter levels, lowering proinflammatory cytokines such as TNF-α and IL-6, and reducing corticosterone elevation (Chandrasekhar et al., 2017). Another key component, Lion’s Mane mushroom extract may have contributed to these behavioral improvements through its stress-reducing and cognition-enhancing properties. In a randomized, placebo-controlled human study (Docherty et al., 2023), reported that both acute and chronic supplementation with *H. erinaceus* improved task performance speed and tended to reduce perceived stress, suggesting that its bioactive compounds may promote adaptive stress responses and exploratory activity. Additionally, Rhodiola rosea, another major constituent of the supplement, has been shown to attenuate corticosterone elevation and modulate stress-responsive gene expression in the hippocampus and prefrontal cortex, thereby reducing behavioral reactivity to acute stress (Dinel et al., 2019). Likewise, Roohi-Azizi et al. (2018) demonstrated that citicoline administration improved open field performance in chronically stressed rodents, reflecting enhanced exploratory behavior and reduced anxiety when co-administered with antidepressant treatment (Roohi-Azizi et al., 2018). Since the present study tested only the combined formulation, we cannot disentangle the contribution of each component. However, these findings suggest that the supplement alleviates stress-induced anxiety and promotes exploratory motivation by synergistically regulating neuroinflammatory, monoaminergic, and neurotrophic pathways, supporting a more stable and adaptive behavioral profile under chronic stress conditions.

In the forced swim test, Mind Focus supplementation significantly reduced immobility time compared with the stress group, suggesting an antidepressant-like effect under chronic stress conditions. This behavioral improvement is consistent with the known neurochemical and neurotrophic actions of several key components within the formulation, although we cannot disentangle the contribution of each component. L-theanine has previously been shown to ameliorate depressive-like behavior in CUMS models by enhancing serotonergic, dopaminergic, and noradrenergic transmission in the prefrontal cortex and hippocampus, accompanied by reduced plasma corticosterone levels (Shen et al., 2019). Similarly, Rhodiola rosea extract has been demonstrated to reduce corticosterone elevation and modulate stress-responsive gene expression in limbic regions, thereby normalizing HPA axis reactivity and improving mood stability (Dinel et al., 2019). Citicoline has also been reported to potentiate antidepressant efficacy by improving sucrose preference, locomotor activity, and behavioral resilience in stressed rodents (Roohi-Azizi et al., 2018), likely through enhanced monoaminergic and membrane phospholipid metabolism. In addition, Ginkgo biloba extract exerts protective effects against chronic stress-induced depressive phenotypes by regulating hippocampal *BDNF* expression and preventing its stress-related methylation-dependent downregulation (Ayatollahi et al., 2020). Together, these findings suggest that the supplementation may counteract the stress-induced depressive behavior by integrating serotonergic, dopaminergic, and neurotrophic mechanisms, maintaining hippocampal plasticity and reducing behavioral despair in response to chronic stress.

Biochemical results indicate that chronic stress induced marked peripheral and central inflammatory activation, particularly reflected by elevated IL-1β, IL-6, IL-10, and TNF-α levels. Mind Focus supplementation, especially in the SMF group, effectively attenuated these changes, maintaining cytokine profiles near control values and suggesting notable immunomodulatory and anti-inflammatory properties. In parallel, the oxidative stress index (TOS/TAS ratio) was markedly increased in both liver and serum of stressed animals, indicating a pronounced systemic oxidative imbalance. The administration of supplement, particularly in the MF and SMF groups, significantly reduced the TOS/TAS ratio toward control levels, demonstrating its antioxidant efficacy and capacity to restore redox homeostasis under chronic stress conditions. These findings are in agreement with previous evidence highlighting the immunomodulatory potential of L-theanine, a key component of the combined formulation, although we cannot disentangle the contribution of each component L-theanine has been shown to regulate immune and oxidative pathways by suppressing the overproduction of pro-inflammatory cytokines such as IL-1β, IL-6, and TNF-α, while enhancing antioxidant defense through glutathione upregulation and free-radical scavenging activity (Chen et al., 2023). Moreover, its ability to attenuate stress-related activation of the hypothalamic–pituitary–adrenal (HPA) axis and inhibit NF-κB signaling contributes to reduced neuroinflammation and improved neuronal resilience under chronic stress. In addition to these mechanisms, Lion’s Mane mushroom extract—also included in the supplement—has been shown in a recent randomized controlled human trial to increase neurotrophic and antioxidant biomarkers, improve psychomotor speed, and reduce subjective stress when taken daily (Contato and Conte-Junior, 2025). Furthermore, *Rhodiola rosea* extract has been reported to exert adaptogenic, anti-inflammatory and antioxidant effects by normalizing stress hormone release, down-regulating stress-responsive cytokines and enhancing resilience to oxidative and inflammatory insult (Pu et al., 2020). Ginkgo biloba extract, another constituent of the supplement, has demonstrated robust neuroprotective and anti-inflammatory effects by improving cerebral microvascular perfusion, attenuating microglial activation and lowering oxidative stress markers in the brain; it has also been shown to modulate both pro-inflammatory cytokines and neurotrophic factors in stress and neurodegenerative contexts (Mousavi et al., 2022). Collectively, these results suggest that the anti-inflammatory and antioxidant effects of the supplement may reflect the combined actions of L-theanine, Lion’s Mane, *Rhodiola rosea* and *Ginkgo biloba*, together with other bioactive compounds, thereby restoring systemic and neural homeostasis disrupted by prolonged stress exposure.

Histopathological evaluation further supports the neuroprotective potential of Mind Focus under chronic stress conditions. Sections from stressed rats displayed characteristic features of stress-related neuronal injury, including triangular perineuronal halos, cytoplasmic eosinophilia, and shrunken “dark” neurons in both the hippocampus and prefrontal cortex. In contrast, tissues from the Stress + Mind Focus group exhibited substantially preserved neuronal morphology with fewer degenerative changes, as reflected by significantly lower semi-quantitative injury scores in both regions. These findings suggest that the supplementation may attenuate the stress-induced neuronal damage, possibly through its combined antioxidant, anti-inflammatory, and neurotrophic actions. Although the contribution of each component cannot be disentangled, a previous study has reported that L-theanine counteracted the oxidative and enzymatic disturbances in the brain by restoring glutathione levels, normalizing membrane-bound ATPase activity, and reversing cortical and hippocampal histopathological alterations in toxin-induced injury models (Sumathi et al., 2015). In the literature, the components such as L-theanine and Ginkgo biloba have been shown to reduce neuronal apoptosis, suppress NF-κB–mediated microglial activation, and preserve synaptic integrity under oxidative or glucocorticoid stress (Chandrasekhar et al., 2017; Chen et al., 2023; Nguyen and Alzahrani, 2023; Shen et al., 2019; Sumathi et al., 2015; Wang et al., 2022; Weinmann et al., 2010; Yeh et al., 2015), while *Rhodiola rosea* and Lion’s Mane extracts enhance neurogenesis and neuronal resilience through BDNF-linked signaling pathways (Dinel et al., 2019; Ganzera et al., 2001; Marchev et al., 2016). Recent evidence indicates that Lion’s Mane mushroom extract also attenuates neuroinflammatory responses and oxidative stress by downregulating proinflammatory cytokines, upregulating antioxidant enzymes such as SOD and catalase, and promoting synaptic plasticity and memory-related signaling in the hippocampus (Zhang et al., 2020). Moreover, *Rhodiola* extract has been shown to exert strong neuroprotective and anti-apoptotic effects by preserving mitochondrial integrity, restoring ATP and cytochrome c oxidase levels, and preventing hippocampal neuronal apoptosis in experimental models of neurodegeneration and chronic stress (Wang et al., 2017). Similarly, phosphatidylserine supplementation has been demonstrated to reduce hippocampal TNF-α and IL-10 expression, enhance neuronal survival, and alleviate depression-like behaviors through its anti-inflammatory and neuroprotective properties in post-stroke models (Partoazar et al., 2021). Together, these effects likely contribute to the observed morphological protection in cortical and hippocampal circuits.

In the present study, hippocampal and prefrontal expression analyses revealed that CUMS induced significant upregulation of *FOS*, *DBH*, and *NMB*, while Mind Focus supplementation was associated with non-significant trend toward control-like values, and with a significant reduction in *NMB* expression compared to stressed animals. These findings are consistent with the hypothesis that chronic stress activates hippocampal stress-responsive transcriptional programs and noradrenergic signaling, which can be modulated by neuroactive nutritional interventions.

*FOS* is an immediate-early gene that serves as a marker of neuronal activation and stress responsiveness. Its upregulation in the hippocampus following chronic stress exposure aligns with previous reports demonstrating increased neuronal activity in stress-related circuits and impaired inhibitory regulation under prolonged stress conditions. Lower *FOS* expression observed in the Mind Focus-treated groups relative to the stress group suggests a potential dampening of stress-induced hyperactivation, possibly through anxiolytic and neuroregulatory effects of L-theanine, phosphatidylserine, and *Rhodiola* extracts, which have been shown to modulate HPA axis reactivity and glutamatergic signaling. Although the contribution of each component cannot be disentangled, a previous study by Kawada et al. has reported that L-theanine suppresses NMDA-induced c-Fos expression and inhibits mTOR-p70S6K pathway activation in the hippocampus, thereby reducing excitotoxic neuronal activity and contributing to its sedative and neuroprotective effects (Kawada et al., 2025). Moreover, Wong et al. demonstrated that daily administration of Lion’s Mane mushroom extract enhances functional and structural nerve recovery after peripheral nerve injury by upregulating Akt and MAPK signaling as well as c-Fos expression, suggesting that Lion’s Mane may support neuronal survival and regeneration through similar activity-dependent transcriptional pathways (Wong et al., 2012). Consistently, Xia et al. (2016) found that Rhodiola rosea reduced stress-induced c-Fos expression in the hypothalamus and normalized corticosterone and CRH levels, indicating that its adaptogenic and anti-stress effects may partly operate via downregulation of stress-responsive immediate-early genes (Xia et al., 2016). In addition, Bennett and Semba (1998) reported that caffeine induces region-specific and dose-dependent changes in c-Fos expression, with low stimulant doses enhancing locomotion without widespread neuronal activation, and higher doses triggering marked c-Fos induction in striatal, limbic, and hypothalamic regions, suggesting a nuanced, dose-regulated modulation of neuronal excitability (Bennett and Semba, 1998). Collectively, these findings suggest that the combined action of Mind Focus constituents, from literature-based hypotheses about individual components, may contribute to the neuronal excitability and may limit maladaptive stress-induced transcriptional activation, leading to a more balanced neural network response that supports behavioral stability, emotional regulation, and stress resilience under chronic stress conditions.

*DBH*, the rate-limiting enzyme in the conversion of dopamine to norepinephrine, serves as a key marker of catecholaminergic turnover and stress responsiveness. In our study, hippocampal and prefrontal *DBH* expression was significantly elevated in the stress group compared to controls, consistent with hyperactivation of noradrenergic circuits under chronic stress. Mind Focus supplementation was associated with *DBH* expression levels closer to control group in both regions, suggesting a stabilizing effect on catecholaminergic neurotransmission. This modulation could plausibly involve combined effects of its bioactive components; however, attribution to a specific component is not possible. Previous studies reported that the caffeine, L-theanine, and citicoline independently influenced the dopaminergic–adrenergic balance. Specifically, Kim (2002) demonstrated that caffeine, through antagonism of adenosine A1 and A2A receptors co-localized with dopamine D1 and D2 receptors in striatal neurons, enhances dopaminergic signaling and restores locomotor and feeding behavior even in dopamine-depleted (Dbh-/-) mice (Kim, 2002). Within this framework, our findings are consistent with the notion that adenosine receptor blockade by caffeine facilitates downstream dopaminergic and noradrenergic activation, potentially attenuating stress-induced catecholaminergic dysregulation, although no statistically significant normalization was demonstrated. Additionally, Lion’s Mane mushroom extract has been shown to enhance dopaminergic function and neuronal plasticity by promoting neurotrophin-mediated survival pathways, particularly via activation of Akt and MAPK cascades (Wong et al., 2012). These mechanisms not only support catecholaminergic neuron integrity but also facilitate adaptive neuronal signaling under stress. *Rhodiola rosea*, a well-characterized adaptogen, has similarly been reported to regulate catecholamine metabolism by reducing corticosterone-induced overactivation of the HPA axis and restoring monoamine turnover in stress-exposed rodents (Dinel et al., 2019). Although the contribution of each component cannot be disentangled, these findings suggest that the combined effects of caffeine, *H. erinaceus*, and *R. rosea* may act to stabilize dopamine–norepinephrine balance through both receptor-mediated and neurotrophic pathways, thereby mitigating stress-related catecholaminergic overdrive and contributing to improved behavioral stability and emotional regulation. Factorial designs (single-ingredient arms and their combinations) or head-to-head comparisons would be required to determine whether any specific component drives this transcriptional pattern or whether synergistic interactions are involved.

*NMB*, a bombesin-like neuropeptide implicated in stress, thermoregulation, and emotional processing, exhibited robust upregulation in the hippocampus under chronic stress. The significant reduction of *NMB* expression in the Stress + Mind Focus group offers a possible mechanistic hypothesis that the supplementation may partially modulate the stress-related peptidergic signaling. Although the contribution of each component cannot be disentangled, this effect may be mediated through indirect modulation of limbic circuits and neuroendocrine feedback mechanisms, consistent with previous evidence linking nutritional bioactives such as *Ginkgo biloba* and *Rhodiola rosea* to the regulation of neuropeptide expression and neuronal plasticity under stress. In line with these findings, Williams and Naidoo (2020) highlighted that endoplasmic reticulum (ER) lipid homeostasis is crucial for neuropeptidergic vesicle production and circadian regulation (Williams and Naidoo, 2020). They demonstrated that ER lipid defects in neuropeptidergic neurons disrupt phosphatidylserine metabolism, impairing vesicular neuropeptide transport and contributing to stress- and sleep-related neuronal dysfunction. Remarkably, phosphatidylserine supplementation restored neuropeptide vesicle formation and normalized behavioral rhythms in Drosophila models. Our findings offer a possible mechanistic hypothesis suggesting that components of the supplement may restore neuropeptidergic balance under chronic stress by improving ER homeostasis and maintaining vesicular signaling integrity, ultimately supporting adaptive emotional and physiological regulation.

Chronic stress is well known to suppress neuroplasticity-related signaling in corticolimbic circuits, leading to structural and functional impairments associated with anxiety, anhedonia, and cognitive decline. *Ginkgo biloba, Rhodiola rosea*, and Lion’s Mane mushroom extract were previously reported to enhance *BDNF* expression and *CREB* phosphorylation through modulation of MAPK and PI3K/Akt pathways, thereby promoting neuronal survival and synaptic remodeling under stress conditions (Ayatollahi et al., 2020; Wang et al., 2017; Wong et al., 2012). In the present study, hippocampal and prefrontal *BDNF* and *CREB1* expression exhibited a downward trend under chronic stress, consistent with impaired neurotrophin signaling and transcriptional regulation of synaptic genes. Mind Focus supplementation suggesting a restorative effect on *BDNF* and *CREB1* expressions at control-like levels, suggesting a non-significant trend toward control-like values on neurotrophic and transcriptional homeostasis. However, these differences did not reach statistical significance and should therefore be interpreted as trends rather than demonstrated effects.

It should also be acknowledged that the qPCR panel comprised multiple stress- and neuroplasticity-related genes assessed across two brain regions, increasing the potential for false-positive findings despite *post-hoc* corrections. Accordingly, the molecular analyses were conducted in an exploratory framework. Genes showing consistent stress-related changes with clear statistical support—such as *FOS* and *NMB* in the hippocampus and *BDNF* in the prefrontal cortex—may be considered more robust findings, whereas non-significant or trend-level changes should be interpreted as hypothesis-generating and warrant validation in independent cohorts or targeted follow-up studies.

*GRIN2A*, a key NMDA receptor subunit critical for excitatory neurotransmission and synaptic plasticity, showed mild downregulation under stress with no significant differences between stress-induced and Mind Focus treated rats. This finding counteracts the stress-induced transcriptional dysregulationon glutamatergic activity mediated by L-theanine and citicoline, which have been shown to regulate glutamate–GABA balance and protect against excitotoxicity via NMDA receptor modulation (Chandrasekhar et al., 2017; Roohi-Azizi et al., 2018). On the other hand, the significant reduction in hippocampal *GABRB1* expression observed in the stress group reflects weakened inhibitory GABAergic tone, a hallmark of chronic stress–induced hyperexcitability. The supplementation did not significantly restore but sustained hippocampal *GABRB1* expression compared to the stress-induced rats, indicating that possible modulation of inhibitory signaling that may underlie its anxiolytic and mood-stabilizing behavioral effects observed in the open field, elevated plus maze, and forced swim tests. Collectively, these findings suggest that the combined formulation exerts neuroprotective and neuroregulatory actions by sustaining the expression of key genes governing neurotrophic support, excitatory–inhibitory balance, and synaptic adaptability. Through the combined effects of its bioactive constituents—including L-theanine’s glutamatergic modulation, phosphatidylserine’s membrane-stabilizing role, citicoline’s enhancement of phospholipid turnover, and Lion’s Mane mushroom extract and *Rhodiola rosea*’s neurotrophic actions—the supplement appears to mitigate stress-induced transcriptional dysregulation within hippocampal and prefrontal networks, thereby promoting neural resilience, exploratory and emotional stability.

Despite the comprehensive multimodal design integrating behavioral, biochemical, histopathological, and molecular endpoints, several methodological limitations should be acknowledged. First, the use of a single dose and treatment duration (130 mg/kg/day for 30 days) restricts the ability to establish dose–response relationships and optimal treatment windows for Mind Focus. In addition, because only the combined formulation was evaluated, the study cannot determine the relative contribution of individual ingredients or potential synergistic interactions. Another limitation is that only male Wistar rats were included. Sex differences in stress responsivity, neuroinflammatory signaling, and behavioral responses to psychotropic and nutraceutical interventions are well documented. Therefore, the present findings may not fully generalize to females, and future studies should explicitly examine potential sex-dependent effects of Mind Focus supplementation using factorial designs (single-ingredient arms and their combinations) or head-to-head comparisons which employ multiple dosage regimens and post-stress intervention models to dissect component-specific versus synergistic effects. Second, brain tissues were snap-frozen at –80°C prior to fixation for histological analysis. While this approach preserved molecular integrity for gene expression assays, it may have introduced freezing artifacts such as vacuolation, perineuronal halo formation, or dark neuron appearance, potentially confounding morphological interpretation. Although freezing-related artifacts such as vacuolization, perineuronal halos, and dark neuron appearance may affect absolute morphological interpretation, all experimental groups were processed using identical tissue handling and blinded scoring procedures; therefore, relative differences in histopathological injury scores between groups remain informative. For more reliable neuropathological evaluation, future studies should prioritize immediate perfusion or immersion fixation and complement hematoxylin–eosin staining with neurodegeneration and synaptic integrity markers (e.g., NeuN, Fluoro-Jade C, cleaved caspase-3, synaptophysin, MAP2). Third, behavioral assessments were limited to validated paradigms (SPT, OFT, EPM, and FST), which primarily reflect affective and exploratory dimensions. Although these tests collectively capture stress-induced behavioral alterations, they do not provide direct measures of attention, learning, or executive function. Accordingly, any interpretation of potential effects on focus or attentional performance should be considered hypothesis-generating rather than demonstrated outcomes. Future studies incorporating dedicated cognitive paradigms such as novel object recognition, attentional set-shifting, or the five-choice serial reaction time task would be required to directly assess these domains. However, stress and focus are linked in a “sweet spot” way: a little or moderate stress can sharpen attention, but too much or long-lasting stress tends to scatter it and harm concentration. The type (acute vs. chronic), intensity, and duration of stress all matter (Liu et al., 2020). Acute high stress can disrupt top-down control from the prefrontal cortex, making attention more driven by sudden, emotionally salient stimuli instead of task goals, which increases distractibility (Shields et al., 2016). Experiments show that people under acute stress make more errors on attention tasks that require ignoring distractions and are more likely to mind wander and understand material less well (Sänger et al., 2014). Excessive or prolonged stress, especially with strong anxiety and high cortisol, usually impairs focus, learning, and complex thinking (Liu et al., 2020). In light of these findings, it is thought that Kiperin Mind Focus reduces stress or increases stress tolerance, thereby limiting the negative effects of excessive stress on attention and executive functions. Thanks to this indirect effect, it is anticipated that concentration, focus and cognitive performance can be supported. Fourth, although transcriptional data indicated modulation of key neuroplasticity and stress-related genes, protein-level validation (e.g., Western blot or immunohistochemistry for BDNF, CREB, or GABRB1) was not performed. Consequently, post-transcriptional or translational regulation cannot be excluded. Similarly, neurotransmitter quantification (dopamine, serotonin, glutamate, GABA) and hormonal profiling (corticosterone) were not included, limiting mechanistic linkage between gene expression and functional outcomes. Finally, the study duration captured only short-term neurobehavioral and molecular outcomes following supplementation. Longitudinal investigations incorporating post-treatment withdrawal periods, electrophysiological recordings, and advanced neuroimaging (e.g., MRI volumetry, PET-based neuroinflammation markers) would further clarify the persistence and clinical relevance of Mind Focus-induced neuroprotection.

## Conclusion

This study provides comprehensive evidence that Mind Focus supplementation exerts multifaceted neuroprotective, anti-inflammatory, and stress-buffering effects in rats exposed to CUMS. Across behavioral paradigms, supplementation significantly ameliorated stress-induced anhedonia, anxiety, and behavioral despair, as demonstrated by improved performance in the sucrose preference, open field, elevated plus maze, and forced swim tests. These behavioral outcomes indicate that the combined formulation enhances emotional stability, exploratory motivation, and stress resilience under chronic stress conditions.

Biochemically, chronic stress induced robust systemic and neuroinflammatory activation, reflected by elevated IL-1β, IL-6, IL-10, and TNF-α levels, along with increased oxidative stress index (TOS/TAS ratio). Mind Focus—particularly in its SMF—effectively normalized cytokine and oxidative parameters, supporting robust anti-inflammatory and antioxidant capacities. Histopathological analyses further confirmed structural neuroprotection, with treated groups exhibiting preserved neuronal morphology and significantly lower injury scores in both the hippocampus and prefrontal cortex compared to stressed animals.

At the molecular level, Mind Focus was associated with attenuation of CUMS-induced upregulation of *FOS* and *NMB*, while other neuroplasticity-related genes (*DBH*, *BDNF, CREB1, GRIN2A*, and *GABRB1)* showed no statistically significant differences across groups, despite mean expression values in treated animals being numerically closer to control levels. Collectively, these transcriptional patterns are consistent with the possibility that the combined formulation may contribute to limiting stress-related neuronal hyperactivation and peptidergic dysregulation, while exerting modulatory effects on neurotrophic and inhibitory signaling pathways within corticolimbic circuits under chronic stress conditions.

Together, these effects converge to sustain synaptic integrity, maintain redox and neurotransmitter balance, and protect neural circuits from chronic stress–induced degeneration. In summary, Mind Focus acts a neuroprotective, mood-stabilizing, and stress-resilience–promoting supplement that preserves neuronal structure and function under chronic stress conditions. These findings highlight its potential relevance as a nutraceutical strategy for mitigating the neurobehavioral consequences of sustained psychological stress and supporting stress adaptation and emotional regulation, and mental wellbeing. It is believed that Kiperin Mind Focus may indirectly support attention, concentration and focus by reducing stress or increasing stress tolerance. Future research should extend these findings through longitudinal and translational studies exploring dose–response relationships, sex-dependent differences, and the long-term neurobehavioral effects of the supplementation. Integrating advanced imaging, electrophysiological, and proteomic approaches will be crucial to delineate the cellular and circuit-level mechanisms underlying its neuroprotective actions. Moreover, clinical trials in human populations experiencing chronic psychological stress, burnout, or stress-related disorders could clarify its therapeutic potential as a nutraceutical intervention for enhancing stress resilience, emotional resilience, and behavioral stability under stress.

## Data Availability

The raw data for this study can be accessed at https://biruni-university.portaljs.com/@biruni-university/gene-expression-raw-datas.

## Footnotes

1
https://dosecal.cftri.res.in
